# Development of Carboxymethyl Chitosan Nanoparticles Prepared by Ultrasound-Assisted Technique for a Clindamycin HCl Carrier

**DOI:** 10.3390/polym14091736

**Published:** 2022-04-24

**Authors:** Tanpong Chaiwarit, Sarana Rose Sommano, Pornchai Rachtanapun, Nutthapong Kantrong, Warintorn Ruksiriwanich, Mont Kumpugdee-Vollrath, Pensak Jantrawut

**Affiliations:** 1 Department of Pharmaceutical Sciences, Faculty of Pharmacy, Chiang Mai University, Chiang Mai 50200, Thailand; tanpong.c@gmail.com (T.C.); yammy109@gmail.com (W.R.); 2 Plant Bioactive Compound Laboratory (BAC), Department of Plant and Soil Sciences, Faculty of Agriculture, Chiang Mai University, Chiang Mai 50200, Thailand; sarana.s@cmu.ac.th; 3 Cluster of Research and Development of Pharmaceutical and Natural Products Innovation for Human or Animal, Chiang Mai University, Chiang Mai 50200, Thailand; pornchai.r@cmu.ac.th; 4 Cluster of Agro Bio-Circular-Green Industry (Agro BCG), Chiang Mai University, Chiang Mai 50100, Thailand; 5 Division of Packaging Technology, School of Agro-Industry, Faculty of Agro-Industry, Chiang Mai University, Chiang Mai 50100, Thailand; 6 Department of Restorative Dentistry, Faculty of Dentistry, Khon Kaen University, Khon Kaen 40002, Thailand; natthkan@kku.ac.th; 7 Research Group of Chronic Inflammatory Oral Diseases and Systemic Diseases Associated with Oral Health, Faculty of Dentistry, Khon Kaen University, Khon Kaen 40002, Thailand; 8 Laboratory of Pharmaceutical Technology, Faculty II, Berliner Hochschule für Technik (BHT), 13353 Berlin, Germany; vollrath@bht-berlin.de

**Keywords:** carboxymethyl chitosan, experimental design, nanoparticle, ultrasound, clindamycin

## Abstract

Polymeric nanoparticles are one method to modify the drug release of small hydrophilic molecules. In this study, clindamycin HCl was used as a model drug loaded in carboxymethyl chitosan nanoparticles cross-linked with Ca^2+^ ions (CMCS-Ca^2+^). The ultrasonication with experimental design was used to produce CMCS-Ca^2+^ nanoparticles loading clindamycin HCl. The model showed that the size of nanoparticles decreased when amplitude and time increased. The nanoparticle size of 318.40 ± 7.56 nm, decreased significantly from 543.63 ± 55.07 nm (*p* < 0.05), was obtained from 75% of amplitude and 180 s of time, which was one of the optimal conditions. The clindamycin loading content in this condition was 34.68 ± 2.54%. The drug content in nanoparticles showed an inverse relationship with the size of the nanoparticles. The sodium carboxymethylcellulose film loading clindamycin HCl nanoparticles exhibited extended release with 69.88 ± 2.03% drug release at 60 min and a gradual increase to 94.99 ± 4.70% at 24 h, and demonstrated good antibacterial activity against *S. aureus* and *C. acne* with 40.72 ± 1.23 and 48.70 ± 1.99 mm of the zone of inhibition at 24 h, respectively. Thus, CMCS-Ca^2+^ nanoparticles produced by the ultrasound-assisted technique could be a potential delivery system to modify the drug release of small hydrophilic antibiotics.

## 1. Introduction

**Figure 1:**
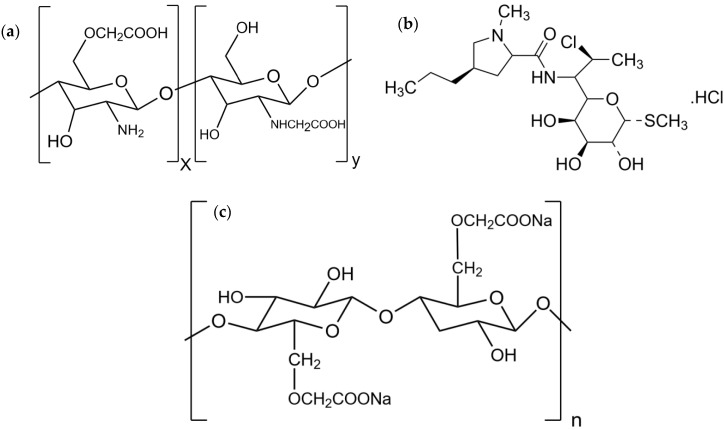
Chemical structure of carboxymethyl chitosan (CMCS) (**a**); clindamycin HCl (**b**); and sodium carboxymethyl cellulose (SCMC) (**c**).

Presently, nanotechnology is one of the most interesting trends for several industries, such as pharmaceutical and cosmetic. Polymeric nanoparticles are normally defined as particles in a range of 10–1000 nm. The polymeric nanoparticle can be loaded with active compounds by an entrapment within or surface-adsorption onto the polymeric core [1,2,3]. Compared with conventional dosage forms, nanoparticles are used to improve drug solubility, increase drug stability, design drug-target delivery and modify controlled release [4]. The polymeric nanoparticle could be prepared by natural polymers, such as alginate, chitosan, collagen, and cellulose, and/or synthetic polymers, such as polylactic acid (PLA) and polyglycolic acid (PGA). However, natural polymers are outstanding due to the biocompatibility and biodegradability. Chitosan is a linear natural polymer obtained from deacetylation of chitin. It consists of d-glucosamine and N-acetyl-d-glucosamine subunits [5]. It is a material of choice for nanoparticle preparation because it possesses suitable properties, for example, biodegradability, biocompatibility, nontoxicity, and ability for physical and chemical modification [6]. Carboxymethyl chitosan (CMCS) consists of active hydroxyl (–OH), carboxyl (–COOH), and amine (–NH_2_) groups in the molecule (Figure 1a). Carboxyl groups refer to the amino and/or hydroxyl groups in chitosan molecules to enhance the solubility in water around neutral pH. CMCS has a wide range of applications in adsorption, drug delivery, biomedicine, cosmetics, food preservatives, biosensors, and emulsion stabilization [7]. At pH ≥ 7, the carboxylic groups (–COOH) in CMCS molecules were deprotonated and changed to anion form (–COO^−^) that can interact with calcium ion (Ca^2+^) to form particles. The suitable pH to produce CMCS cross-linked with Ca^2+^ (CMCS-Ca^2+^) nanoparticles was 7–9. In addition, CMCS-Ca^2+^ nanoparticles were most stable at pH 7–8 and could be applied for controlled release substances [8]. Nano-scale particles could be prepared by ionic-crosslinking, such as CMCS-Ca^2+^ crosslinking and covalent-crosslinking. The drawback of the covalent bonding is potentially the toxic residue due to unreacted covalent-crosslinking agents. To avoid this problem, non-toxic cross-linkers, such as salts as well as Ca^2+^, may be used as ionic cross-linkers. Furthermore, the medium of CMCS-Ca^2+^ nanoparticles is water, which is totally non-toxic. Some studies applied CMCS-Ca^2+^ nanoparticles to entrap active substances for controlled release. For example, the CMCS-Ca^2+^ nanoparticles loading doxorubicin HCl showed suitable size, narrow size distribution, potential drug entrapment, and controlled release [9]. In another study, the nanoparticles loading curcumin, prepared by CMCS-Ca^2+^ cross-linking, exhibited spherical nanoparticles with a mean diameter of 150 nm, high drug loading, controlled release, and non-toxicity property [10]. There are several tools which could aid in producing nanoparticles. Ultrasound is a simple, fast, green, and reproducible method to produce nanoparticles [11]. Ultrasound is longitude wavelength in a frequency of 20 kHz–10 MHz. In ultrasonication, the main phenomenon is acoustic cavitation, which creates, grows, and collapses bubbles in less than nanoseconds [12]. Generally, nanoparticles are required to be in a well-dispersed state. Usually, conventional technologies such as stirred media mills to disperse particles into liquids is insufficient, because the nanoparticles strongly agglomerate; thus high specific energy inputs are required to overcome the adhesive forces. Compared with conventional techniques, ultrasound is one way to break down the agglomeration of nanoparticles in aqueous dispersions [13]. The ultrasound was successfully used for preparing polymeric nanoparticles, such as poly (lactic-co-glycolic acid), starch, chitosan and chitosan- pentasodium tripolyphosphate (TPP) nanoparticles [14,15,16,17]. The studies reported that the particle size and polydispersity index after chitosan nanoparticle preparation with ionic cross-linking could be decreased by increasing sonication time and radiation amplitude [15].

Clindamycin is a small antibacterial molecule and an effective antibacterial agent against several pathogens, such as *Staphylococci*, *Streptococci*, *Pneumococci*, most anaerobic bacteria, *Chlamydia trachomatis*, and some protozoa. Clindamycin is a bacteriostatic antibacterial agent with inhibiting 50 s ribosomal activity. This drug’s mechanism inhibits bacterial protein synthesis. Clindamycin is used for the treatment of skin and soft tissue infections [18]. Thus, it is usually used for topical antibacterial application. Clindamycin is practically insoluble in water; however, clindamycin hydrochloride (HCl) is a freely soluble salt form of clindamycin (Figure 1b). Hence, in this study, clindamycin HCl was selected and used as a model drug of small hydrophilic antibacterial molecules, such that drugs naturally could not provide sustained release patterns [19]. Furthermore, a hydrophilic antibiotic usually exhibits fast release, and the drug concentration, which should be over the minimum inhibitory concentration (MIC), thus decreases. A lower concentration of the drug’s MIC can induce antibacterial resistance and users have to take medicines several times per day to keep the level of drug concentration, which might decrease patient compliance [20]. Extended release of the drug has a potential to resolve this problem. Thus, many drug carriers to provide extended antibiotic release have been developed for antibiotic delivery. In recent years, the topical delivery of antibiotics in the form of nanoparticle drug carriers has demonstrated the efficacy to cure bacterial infections. Nanoparticles are a promising drug delivery system that facilitates the extended release of antibiotics [21]. In addition, encapsulation of the drug in a nanoparticle could also enable the extended release of hydrophilic antibiotics [19,22].

Sodium carboxymethyl cellulose (SCMC) is a semi synthetic ether cellulose derivative with carboxymethyl substitution (Figure 1c). It is commonly used in oral and topical pharmaceutical formulations and in antibacterial applications with high drug loading capacity as hydrogel and film formulation. Moreover, SCMC is able to immobilize *Pseudomonas aeruginosa* and *Staphylococcus aureus* within its cohesive gel structure. Hydrophilic films can be used for the local delivery of active agents such as antimicrobials substances loaded in nanoparticles for infection prevention and treatment [23,24]. SCMC films containing various nanoparticles were also used for an antibacterial application and they showed potential antibacterial activity [23,25]. In this present study, clindamycin HCl-loaded in CMCS cross-linked with Ca^2+^ nanoparticles were prepared using the ultrasound-assisted technique. The CMCS- Ca^2+^ nanoparticle containing clindamycin HCl was loaded into SCMC film. This study aimed to investigate the optimal condition and effect of ultrasonication to reduce particle size, drug release, and antibacterial property of the SCMC film containing the nanoparticles, and also evaluate the possibility of CMCS to be a carrier for extended-release of small hydrophilic antibacterial molecules for an antibacterial application.

## 2. Materials and Methods

### 2.1. Materials

Calcium chloride (CaCl_2_) was purchased from Merck (Damstadt, Germany). Carboxymethyl chitosan (>98% purity, 90% degree of deacetylation) was purchased from Santa Cruz Biotechnology (Dallas, TX, USA). Clindamycin hydrochloride (HCl) was purchased from Merck (Damstadt, Germany). Disodium hydrogen phosphate (Na_2_HPO_4_), sodium chloride (NaCl), and potassium phosphate monobasic (KH_2_PO_4_) were purchased from RCI Labscan Limited (Bangkok, Thailand). Polyethylene glycol 1500 was purchased from Clariant Produkte GmbH (Gendorf, Germany). Potassium chloride was purchased from Kemaus chemical Ltd. (Cherrybrook, Australia). Sodium carboxymethylcellulose (SCMC) was purchased from Kima Chemical (Shanghai, China). Sodium hydroxide (NaOH) and hydrochloric acid (HCl) were purchased from Ajax Finechem (Victoria, Australia). All other reagents were analytical grade.

### 2.2. CMCS-Ca^2+^ Nanoparticles

The method was adapted form Shi et al. [9]. CMCS 0.5 g was dissolved in 100 mL of deionized water and stirred overnight to obtain 5 mg/mL CMCS solution. Then, the pH of the CMCS solution was adjusted to 8 by 0.1 N NaOH. Clindamycin HCl 1.1 g (equivalent to clindamycin base 1 g) was completely dissolved in 100 mL of the CMCS solution. A crosslinking solution was prepared by dissolving CaCl_2_ 3 g in 100 mL of deionized water to obtain 30 mg/mL of CaCl_2_ solution. The ratio between CMCS and CaCl_2_ was 1 mg: 0.0035 mmol. The CaCl_2_ solution 1.3 mL (0.35 mmol of CaCl_2_) was dropped into 20 mL of the CMCS solution (100 mg of CMCS) at room temperature during stirring with a magnetic stirrer at 200 rpm. The solution changed to milk light color simultaneously and the system was stirred continuously for 1 h. The suspension was treated by the ultrasonic probe (UP200ht ultrasonic processor, Hielscher Ultrasonics GmbH, Teltow, Germany) at 26 kHz. The 30 mL solution in a polyethylene container was sonicated at room temperature in 80% continuous mode of ultrasonication. The probe was immersed 0.5 cm into the sample. The time and amplitude were varied from 60 to 300 s and 40% to 80%, respectively. Each experiment was repeated 3 times. The CMCS-Ca^2+^ nanoparticles were measured for the particle size immediately. The CMCS-Ca^2+^ nanoparticles were dried by lyophilization and kelp in an air-tight container at room temperature, respectively.

### 2.3. Experimental Design

The conditions of the ultrasound-assisted technique to produce CMCS-Ca^2+^ nanoparticles were designed by central composited design (CCD) using Design-Expert^®^ software version 12 (Stat-Ease, Inc., Minneapolis, MN, USA). The variable factors were the amplitude of ultrasonic probe and time. Each variable factor contained 3 levels of experiments (Table 1). The amplitude and ultrasonication time were varied from 40% to 80% and 60–300 s. The response surface was displayed as a contour plot, a 3D graph of amplitude and ultrasonication time versus one particle size. Each designed experiment, to prepare a predictable model, was repeated 3 times. Statistical analysis of the difference between predicted and actual values was performed using a *t*-test. Predictability of the obtained model was computed and evaluated by the software.

### 2.4. Particle Size Measurement

The diameter of the nanoparticles was measured immediately after ultrasound preparation by Zetasizer (Zetasizer 3000, Malvern, Kassel, Germany). The sample was not diluted before measurement and viscosity of dispersant was set as water at 25 °C.

### 2.5. Lyophilization

Lactose was added into nanosuspensions in concentration of 1% *w*/*v* to be a filler and a cryoprotectant. The nanosuspensions (20 mL) were transferred to a 50 mL centrifuge tube and placed in a refrigerator at −20 °C for 48 h. Afterward, lyophilization was carried out in a freeze dryer (Beta 2–8 LSCbasic, Martin Christ Gefriertrocknungsanlagen GmbH, Osterrode am Harz, Germany) for 72 h under vacuum pressure of 0.1 mbar and a condenser temperature of −80 °C. The dry powder was kept in a refrigerator at 2–8 °C before loading into the SCMC film.

### 2.6. Fourier-Transform Infrared Spectroscopy

Chemical interactions and structures of CMCS and clindamycin in CMCS-Ca^2^+ nanoparticles were investigated by Fourier transform infrared (FTIR) spectrometer (FT/IR-4700, Jasco, Tokyo, Japan). The samples were scanned from 400–5000 cm^−1^ at a resolution of 4 cm^−1^ in transmittance mode.

### 2.7. Drug Loading Content in CMCS-Ca^2+^ Nanoparticles

The method to determine drug content in CMCS-Ca^2+^ was adapted from Shi et al. [Shi-9]. The clindamycin HCl loading content in the CMCS-Ca^2+^ nanoparticles was determined by indirect method. The CMCS-Ca^2+^ nanoparticle containing clindamycin (10 mL) was precipitated by centrifugation at 6000 rpm for 3 h. The aliquoted supernatant of 1 mL was diluted to 10 mL using a phosphate buffer saline (PBS) pH 7.4. Then, the samples were filtered by a 0.2 micropore filter. Clindamycin HCl content in the filtrate was measured by UV-spectrophotometer (UV-2600i, Shimadzu, Kyoto, Japan) at 204 nm and calculated for the drug content using the standard curve prepared with 25–75 µg/mL of clindamycin HCl solution in PBS pH 7.4. The standard curve exhibited high correlation coefficient (R^2^ = 0.996). The drug loading content was calculated by Equation (1)

(1) $$\mathrm{Drug}\;\mathrm{loading}\;\mathrm{content}\;(\%)=\frac{Theorectical\;drug-Amount\;of\;drug\;in\;supernatant}{Theorectical\;drug\;}\times 100$$

### 2.8. Transmission Electron Microscope (TEM) Imaging of CMCS-Ca^2+^ Nanoparticles

The imaging method was described in Kalliola et al. [8]. One drop of the CMCS-Ca^2+^ nanoparticle suspension was dropped on the sample holder grid without any coating solution and placed at room temperature to dry the samples before analysis. The CMCS-Ca^2+^ nanoparticles were imaged by transmission electron microscope (TEM) (JEOL JEM 2010, Japan Electron Optics Laboratory Co., Ltd., Tokyo, Japan). The size and shape of the nanoparticles were evaluated based on the obtained TEM micrographs.

### 2.9. Preparation of Sodium Carboxy Methylcellulose (SCMC) Film Loading CMCS-Ca^2+^ Nanoparticles

The method to prepare polymeric film was adapted from Ghorpade et al. [26]. Briefly, SCMC solution was prepared by dissolving 2 g of SCMC power in 100 mL of distilled water using a magnetic stirrer to obtain 2% *w*/*v*. Two hundred milligrams of PEG1500 (10% based on SCMC mass) and 0.4 g of citric acid were added into the solution. The solution was stirred continuously to obtain a homogeneous solution. Then, 0.4 g of glycerol (10% based on SCMC mass) was added into the solution as an effective plasticizer. After that, 1.1 g of clindamycin HCl powder or 13.35 g of the lyophilized CMCS- Ca^2+^ nanoparticle, which contained clindamycin HCl 1.1 g, was dissolved homogeneously in the solution. Air bubbles in the solution were removed by placing the solution at room temperature overnight. The bubble free solution 9 g was poured into a Petri dish (6 cm of diameter) and dried in a hot air oven to eradicate water at 40 °C for 24 h. The dry film was removed from the Petri dish and kept in silicon paper and aluminum foil.

### 2.10. Scanning Electron Microscope (SEM) Imaging of SCMC film loading CMCS-Ca^2+^ Nanoparticles

Morphology of the SCMC films was observed for their surface characteristics and cross-sections by JEOL JCM-7000 NeoScope™ Benchtop SEM (JEOL, Tokyo, Japan). Prior to imaging, uncoated SCMC film samples were mounted on aluminum stubs using double-sided carbon tape (NEM tape, Nisshin Co., Ltd., Tokyo, Japan), followed by gold sputter coating for 1 min, and then positioned on the stage in the imaging compartment of the device. SEM images of all of the SCMC film samples were collected using a secondary electron detector at a 10 kV acceleration voltage under low vacuum mode. Film surface and cross-sectional images were observed at 500 and 2500 magnification levels, respectively.

### 2.11. Clindamycin HCl Loading Content in the SCMC Film Containing CMCS- Ca^2+^ Nanoparticles

The SCMC film samples (2 cm × 1 cm) were dissolved in 15 mL water with a magnetic stirrer at 500 rpm for 48 h to completely dissolve the films. Then, the pH was adjusted to 5 with HCl 0.1 N. The samples were filtered and diluted with PBS pH 7.4. The content of clindamycin HCl was determined by UV-spectrophotometer (UV-2600i, Shimadzu, Kyoto, Japan) at 204 nm of wavelength. The experiment was replicated 3 times.

### 2.12. Drug Release Profile

The in vitro clindamycin HCl release study was adapted from Junmahasathien et al. [27]. SCMC film containing clindamycin HCl (SCMC-CM-powder) and SCMC film containing CMCS-Ca^2+^ nanoparticles loading clindamycin HCl (SCMC-CM-nano) were cut to a square shape (2 cm × 1 cm) and immersed in 15 mL PBS pH 7.4 at 37 ± 0.5 °C in semi-static conditions. At each predetermined time (1, 5, 15, 30, 60, 120, 240,360, 480, 720, and 1440 min.), 3 mL of the media was taken and replaced with PBS pH 7.4, respectively. The clindamycin in the taken media was analyzed by UV-spectrophotometer (UV-2600i, Shimadzu, Kyoto, Japan) at 204 nm of wavelength. The experiment was replicated 3 independent times.

### 2.13. Antibacterial Activity

Antibacterial activity of the SCMC-CM-powder and SCMC-CM-nano films against *Staphylococcus aureus* and *Cutibacterium acnes* was investigated by disc diffusion assay. The method was described in [28]. Stock cultures of *S. aureus* (ATCC25923) and *C. acnes* (ATCC6919) were prepared by growing in tryptic soy agar (HiMedia, Mumbai, India) at 37 °C for 24 h; subsequently inoculating into tryptic soy broth (Sigma-Aldrich, St. Louis, MO, USA) and growing at 37 °C under aerobic atmosphere for 12 h. The optical density at 600 nm (OD600) of the growth cultures of *S. aureus* and *C. acnes* were measured by spectrophotometer (Beckman Coulter, Fullerton, CA, USA) to validate for a fresh preparation of bacterial stocks. All film samples were sterilized by ethylene oxide prior to an antibacterial test. The antibacterial activity against *S. aureus* and *C. acnes* was investigated by the agar diffusion method with clindamycin content in the film samples. In brief, 100 µL bacterial stock (OD600 = 0.1) was plated on a dried tryptic soy agar plate and let dry. The tested circle films (0.9 mm diameter) were placed on the prepared agar plate and incubated at 37 °C in an aerobic incubator for 24 h. The antibacterial activity of the SCMC film samples was observed in terms of zone of inhibition (clear zone), measured by Mitutoyo^®^ Digimatic caliper (Mitutoyo Corporation, Kanagawa, Japan). The zone of inhibition was measured at 3, 6, 9, 12, and 24 h after placing the samples. Bacterial growth under the film discs was also investigated. The SCMC film without any clindamycin HCl was used as a blank control. The positive control was clindamycin HCl 1% *w*/*v*, loaded in a Whatman^®^ antibiotic assay disc (GE Healthcare, Pittsburgh, PA, USA).

### 2.14. Statistic Analysis

The analyzed data was presented as mean ± standard deviation (S.D.). The SPSS software (version 17; IBM corporation, New York, NY, USA) was used to analyze a significant difference of analyzed results. A significant level was set as 0.05 (*p* < 0.05) to be considered statistically different. The Design-Expert program (version 12; State-Ease Inc., Minneapolis, MN, USA) was used to design the predictable model and evaluate the model’s qualities in the experimental design.

## 3. Results and Discussion

### 3.1. Nanoparticle Preparation Using the Ultrasound-Assisted Technique

CMCS-Ca^2+^ nanoparticles were formed by ionic interaction between carboxylic anion (-COO^−^) and Ca^2+^. The pH of the CMCS solution was 8. In this pH, carboxylic groups are in anion forms that are suitable to interact with Ca^2+^ in the solution [8]. The pH of the solution during preparation is a crucial factor to determine particle size. At a pH of 7–8, the CMCS-Ca^2+^ is most stable where CMCS is in negative charge (-COO^−^) as majority and in positive charge (NH_3_^+^) as minority. Thus, the CMCS could interact with Ca^2+^ to form nanoparticles and the positive charge in molecules prevents agglomeration. In addition, a concentration of CaCl_2_ seems to be essential since particle size tended to be larger when Ca^2+^ increased in the system [8]. These factors should be controlled because they affect the stability, degradation, and drug release. The CMCS-Ca^2+^ containing clindamycin HCl without an ultrasound-assisted treatment exhibited around 543.63 nm mean particle diameter. On the other hand, the CMCS-Ca^2+^ containing clindamycin HCl treated by ultrasonication showed a particle size around 320 to 490 nm. It indicated that ultrasonication can reduce particle size of CMCS-Ca^2+^ containing clindamycin HCl. The previous study also found that the ultrasound-assisted technique can decreased mean particle diameter [15].

### 3.2. Experimental Design

**Figure 2:**
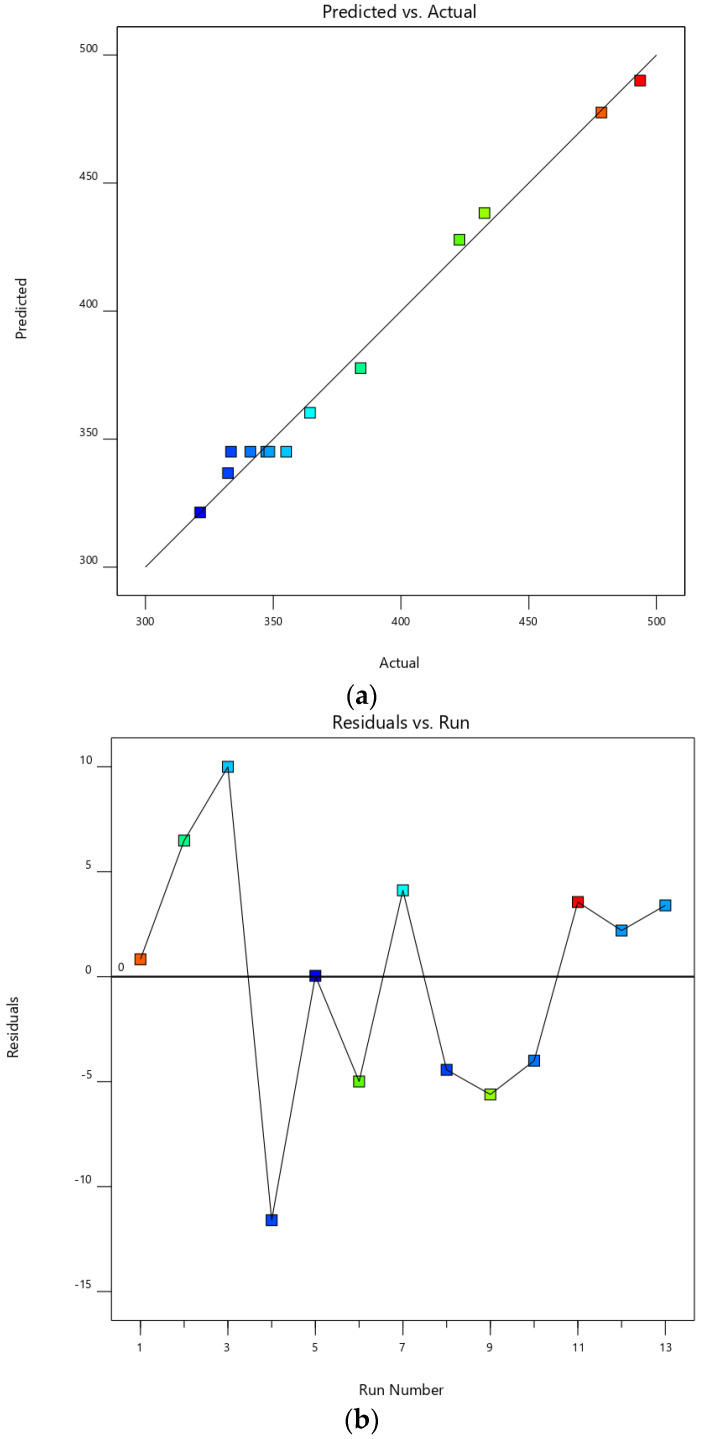
The plot between actual and predicted values (**a**) and the plot between residual and run (**b**).

The quadratic model was suitable to predict particle size with higher adjusted and predicted R^2^ along with non-significant lack of fit (*p* > 0.05) (Table 2). The significance of F-value of the model implies that there is only a 0.01% chance that standard deviation could occur due to noise. The result obtained from analysis of variance (ANOVA) showed a statistically significant difference of coefficients of amplitude and time between groups (*p* < 0.0001), as shown in Table 1. Almost all model terms were significant (*p* < 0.0001) except amplitude × time (AB). There was no significant interaction between amplitude and time. The adjusted R^2^ (0.9820) was a reasonable agreement with predicted R^2^ (0.9627) because the difference between both R^2^ was less than 0.2. The model displayed high adequate precision (32.2313), which is measured by ratio between signal and noise and should be less than 4, indicating the model can be used to navigate the design space. The graph plotted between predicted and actual responses displayed that the model provided the predicted particle size approximately and the actual particle size with high correlation coefficient (R^2^ = 0.9895) of the plot (Figure 2a). The plot between residuals and run number showed an irregular trend (Figure 2b). These results indicated that the model could be used to predict the size of nanoparticles obtained from the ultrasound-assisted treatment. The equation obtained from the model was shown in Equation (2).

Size = +886.72784 − 12.63258 × amplitude − 0.704668 × time − 0.001781 × amplitude × time + 0.085359 × amplitude^2^ + 0.001701 × time^2^ (2)

The size of nanoparticles obtained from various experiments was shown in Table 2. The actual values were not significantly different from the predicted value (*p* > 0.05) in *t*-test. It indicated that the model was accurate and could predict the mean diameter of nanoparticles. From the software, the standard deviation of predicted values was 7.70 nm. The contour plot and 3D response surface plot exhibited effects of amplitude and time on nanoparticle size. In a range of experiments, the contour plot and 3D response surface obviously showed that the size of nanoparticles tended to decrease when amplitude and time increased. This trend corresponded to Tang et al. [15] and Esmaeilzadeh-Gharedaghi et al. [17], summarizing that the use of ultrasound with increasing amplitude and duration could decrease mean diameter of chitosan particles. When ultrasonication is applied, the main mechanism to reduce particle size is the formation of acoustic cavitation, occurring all over the solution. This phenomenon generates very high shear force and breaks the particles into smaller ones possessing fewer chitosan chains in each particle [12]. The effect of amplitude may be also explained by the mechanism of ultrasound. The stronger ultrasound radiation led to smaller particle size due to breaking more particles apart and an increase of the cavitation effect due to longer exposure to ultrasonic radiation. Thus, longer ultrasonication time provides smaller particle sizes. The previous study found that the size of chitosan nanoparticles prepared by ionic cross-linking was reduced with increasing duration of ultrasonication [16]. There were other factors affecting the size of CMCS nanoparticles, for instance the pH of the solution and concentration of chitosan. The pH of CMCS solutions in this study was fixed at 8 because it was the most suitable pH with high amounts of carboxylic anion to interact with Ca^2+^ [8]. In another study, it was found that increasing the concentration of chitosan at fixed duration increased the obtained nanoparticle size. However, when time and amplitude are high enough, the concentration of chitosan has an insignificant effect on the mean diameter of the obtained nanoparticles [17]. These indicated that amplitude and time were the most important input parameters to reduce the size of nanoparticles.

**Figure 3:**
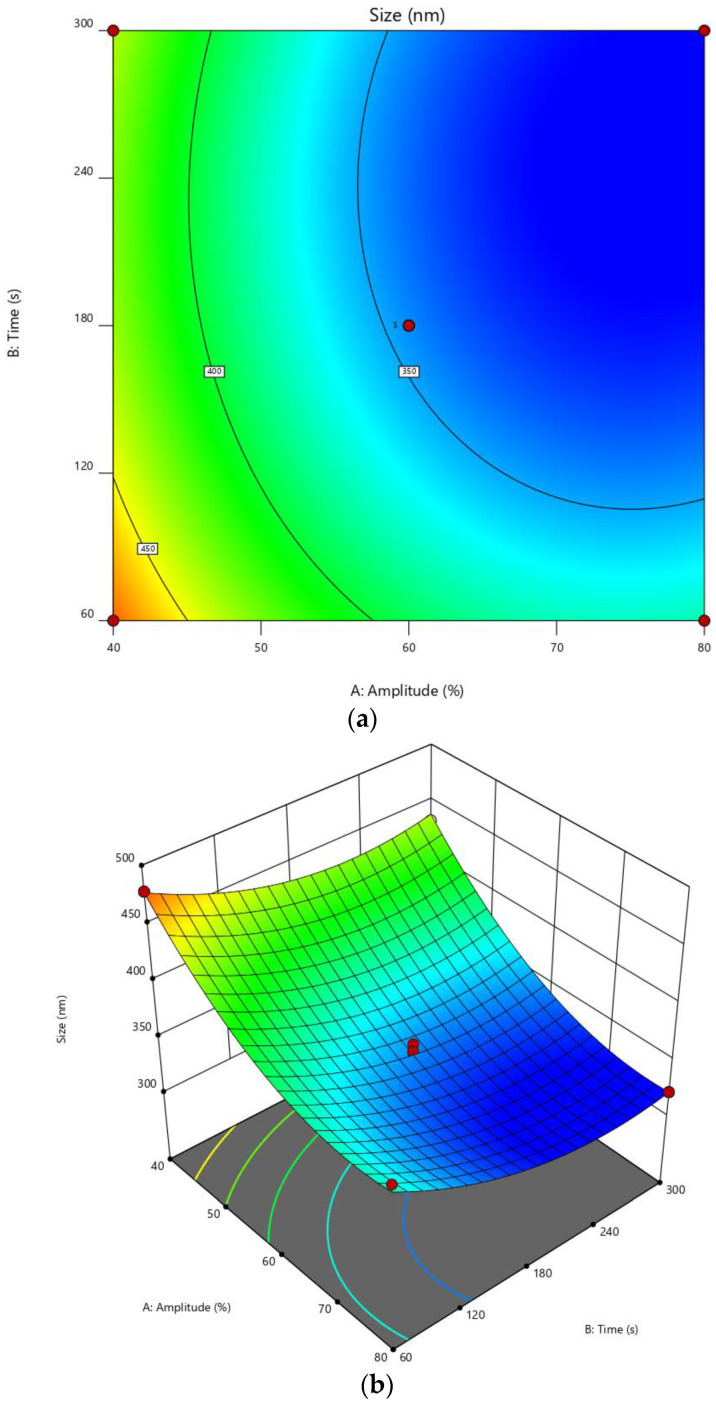
Contour plot (**a**) and 3D response surface (**b**). Blue, light blue, green, yellow, and red colors were 321, 350, 400, 450, and 493 nm, respectively.

In addition, the plateau trend was observed in the blue area of the contour plot and 3D response surface (Figure 3). It showed the minimum size which could be obtained from the ultrasound-assisted technique. In this study, the ultrasound-assisted technique reached a limit to reduce particle size at around 320 nm (blue color in Figure 3). For example, the obtained particle size from 65% of amplitude at 200 s was not significantly different to 75% of amplitude and 180 s of duration (*p* > 0.05) (Table 3). This could be explained by the theory that in chitosan molecules some bonds are weak and broken simply, but other bonds are strong and resistant against applied energies in the experiments [29]. Another reason was the use of Ca^2+^ cross-linker forming polymer chain structures, that prevent breaking particles from becoming smaller ones. Another study also found that the use of tripolyphosphate as the cross-linker for chitosan nanoparticles resisted breaking particles of ultrasonication [17]. Overall, to obtain nanoparticles around the size of 320 nm, the ultrasonication should be performed at a high value of amplitude, such as 70%, and higher intermediate to high duration, such as 180 and 270 s. Thus, in this study, the selected condition obtained from experimental design was 75% of amplitude and 180 s of time providing a particle size of about 318 nm, which was insignificantly different from the predicted value (323.65 nm) (Table 3).

### 3.3. Particle Size of CMCS-Ca^2+^ Nanoparticles

The particle size of CMCS-Ca^2+^ nanoparticles was shown in Table 3. The particle size of all experimental order and from the predictable model in the range of investigation showed that the mean diameter was about 310 to 500 nm. Polymeric nanoparticles were solid or colloidal particles in a range of 10–1000 nm [1,2,3]. Thus, the obtained particles in this study complied with the definition of a polymeric nanoparticle. Meanwhile, the particle size from the non-assisted ultrasound technique was 543.63 ± 55.07 nm. The polydispersity index (PDI) values of experimental order were in a range of 0.2–0.5, while that of the particles obtained from the non-assisted ultrasound technique was 1.000. The PDI of all experiments was less than 0.7, indicating that the size distribution of nanoparticles was suitable. PDI is used to describe the degree of non-uniformity of size distribution of particles; where a value more than 0.7 indicates heterogeneity of particle size distribution [30]. The ultrasound-assisted technique not only decreased particle size, but also increased the uniformity of nanoparticles.

Nanoparticle size has an impact on the chemical properties of nanoparticles. Smaller particles have higher surface energy than larger particles. Thus, smaller particles agglomerate faster than higher particles [31]. However, in our study, the positive charge of -NH_3_^+^ is able to prevent agglomeration with an electrostatic effect. Similarly, increased degradation rates of nanoparticles are dependent on the decrease of particle size because high surface area to volume ratio contributes to a larger portion of the material exposed to media [32,33]. Hence, drug release might be more rapid in smaller nanoparticles. However, degradation and stability of nanoparticles not only depends on particle size, but also molecular weight, chemical structure, glass-transition temperature, degradation products, and crystallinity [31]. Importantly, small nanoparticles are crucial for antibacterial activity because smaller nanoparticles exhibited more effective antibacterial activity than larger particles [34].

### 3.4. Fourier Transform Infrared Spectroscopy

**Figure 4:**
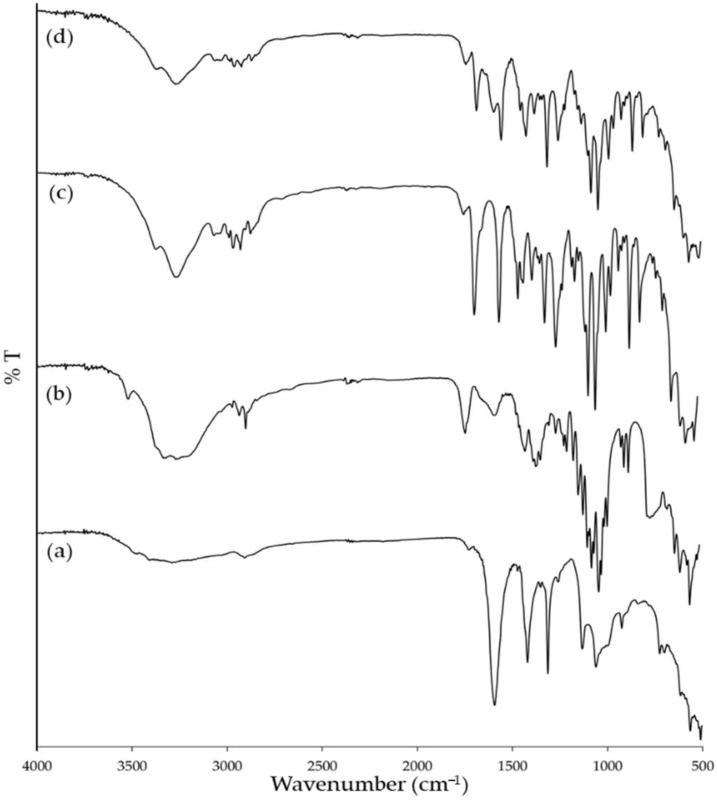
FTIR spectra of CMCS (**a**); CMCS-Ca^2+^ nanoparticle containing clindamycin HCl (**b**); clindamycin HCl (**c**); and physical mixture of CMCS and clindamycin HCl (**d**).

The FTIR spectra of the starting materials (CMCS and clindamycin powders), their physical mixture, and CMCS-Ca^2+^ nanoparticles with clindamycin HCl were shown in Figure 4. In the spectrum of CMCS (Figure 4a), the wide band around 3310 cm^−1^ corresponds to the axial stretching of the O-H and N-H bonds of hydroxy (-OH) and amine (-NH_2_) groups, respectively. The peak at 2900 cm^−1^ relates to the axial stretching of the C-H bond of -CH_2_ group. The weak peak at 1723 cm^−1^ corresponds to the symmetric stretching vibration of C=O in the -COOH groups. The two strong peaks at 1591 and 1417 cm^−1^ are specific for stretching of COO^−^ for carboxylic (-COOH) and for carboxymethyl (-CH_2_COOH) groups, respectively. The peak at 1310 cm^−1^ relates to the symmetric angular deformation of the C-H bond. The peaks in the range of 1129 to 811 cm^−1^ relate to the C-O and C-O-C of the compositions in a polysaccharide chain [35,36,37]. Interestingly, in the spectrum of CMCS-Ca^2+^ nanoparticles containing clindamycin HCl (Figure 4b), the intensity of the peaks corresponding to carboxylic and carboxymethyl at 1591 and 1417 cm^−1^ decreased and both peaks shifted to 1574 and 1421 cm^−1^, respectively. This finding indicated an interaction between -COO^−^ of CMCS and Ca^2+^ in the cross-linking solution [38]. The FTIR spectrum of clindamycin HCl (Figure 4c) showed characteristic peaks of C-H stretching at 2900 cm^−1^, C=O stretching at 1681 cm^−1^, C=C stretching at 1548 cm^−1^, and C-O stretching at 1249 and 1041 cm^−1^ [39,40]. In the spectrum of CMCS-Ca^2+^ nanoparticles containing clindamycin HCl, the characteristic peaks of clindamycin HCl still appeared and slightly shifted to 1215 and 1037 cm^−1^, which indicated that clindamycin HCl was incorporated into the CMCS-Ca^2+^ nanoparticles without changing the general structure. This result was also observed in the FTIR spectrum of clindamycin HCl loaded in polylactic acid (PLA) nanoparticles and in polyvinyl alcohol (PVA) nanofibers, which are hydrophobic and hydrophilic polymers, respectively [39,41]. However, the peak of -OH groups of clindamycin HCl at 3250 cm^−1^ was broader and shifted to 3268 cm^−1^ in the spectrum of CMCS-Ca^2+^ nanoparticles containing clindamycin HCl. Moreover, this region is associated with -OH and -NH_2_ groups of CMCS. In fact, hydrogen bonding formation is marked by band shifting and/or broadening as well as band intensity variation of the relevant bands. A characteristic broad band from 3680 to 2985 cm^−1^ corresponds to different hydrogen-bonded OH groups [42]. Thus, -OH groups in clindamycin HCl molecule possibly interact with hydrophilic groups, such as –OH, to form a hydrophilic interaction or hydrogen bond. The spectrum of the physical mixture (Figure 4d) showed similar peaks to those found in CMCS and clindamycin HCl, such as the peak of carboxylic (-COOH) of CMCS at 1591 cm^−1^, and the peak of C-O stretching of clindamycin HCl at 1249 and 1041 cm^−1^; although the peaks of each starting material overlapped. The spectrum of CMCS-Ca^2+^ nanoparticles with clindamycin HCl was quite similar to the physical mixture, but some peaks altered location as well as intensity, and were broader due to the interactions between CMCS and Ca^2^+, and CMCS and the drug in the CMCS-Ca^2+^ nanoparticles.

### 3.5. Clindamycin HCl Content in CMCS-Ca^2+^ Nanoparticles

Clindamycin HCl loading content is shown in Table 4. The result showed that the drug content was increased when the particle size increased. For example, when the particle size decreased from 478.40 (Exp. 1) to 321.40 nm (Exp. 2), the drug content significantly dropped from 46.88% to 33.30%. This profile was also observed in hyaluronic acid nanoparticle loading clindamycin phosphate. The entrapped clindamycin phosphate content increased with the rise of hyaluronic acid nanoparticles [43]. Previous studies concluded that high intensity ultrasonication damaged chitosan nanoparticles. The damage affected the function of the drug carrier. Both long duration and high intensity could partially depolymerize polymer chains to become smaller fragments [15,44]. Theoretically, the drug with hydrophilic groups could be physically entrapped in a hydrophilic polymer with hydrogen bonding and ionic interaction [9,19]. In this study, clindamycin HCl, containing -OH groups, could form hydrogen bonds with hydrophilic groups of CMCS (-OH and -COOH), but this interaction might not be sufficiently strong enough to resist the ultrasonication. The encapsulated drug in nanoparticles might leak out from the particle structures due to the degradation of nanoparticles and drug–polymer hydrogen bonding. Hence, it was possible that in this study, smaller nanoparticles obtained from higher amplitude and longer duration might exhibit lower drug content. Some factors also influenced the drug content, such as the drug content increased when the molecular weight (MW) of CMCS increased. Less Ca^2+^ is required to form a structure. Therefore, there were more carboxylic groups left for interaction with a drug. Furthermore, compared with the lower MW CMCS, the higher MW with a longer chain could encapsulate drugs with physical interactions, such as adsorption and entrapment. Another factor was a degree of substitution (DS), the higher DS of CMCS had more functional groups to interact with a drug [9]. The drug content in this study was not too low when compared with the study of Shi et al. [9], which exhibited 10–40% doxorubicin content in CMCS-Ca^2+^. Although drug content in the selected experiment (Exp. 16) was 34.68 ± 1.45%, which was lower than the drug content in larger nanoparticles, the smaller nanoparticles tended to exhibit higher antimicrobial effects than the larger nanoparticles [34].

### 3.6. TEM Image of CMCS-Ca^2+^ Nanoparticles

**Figure 5:**
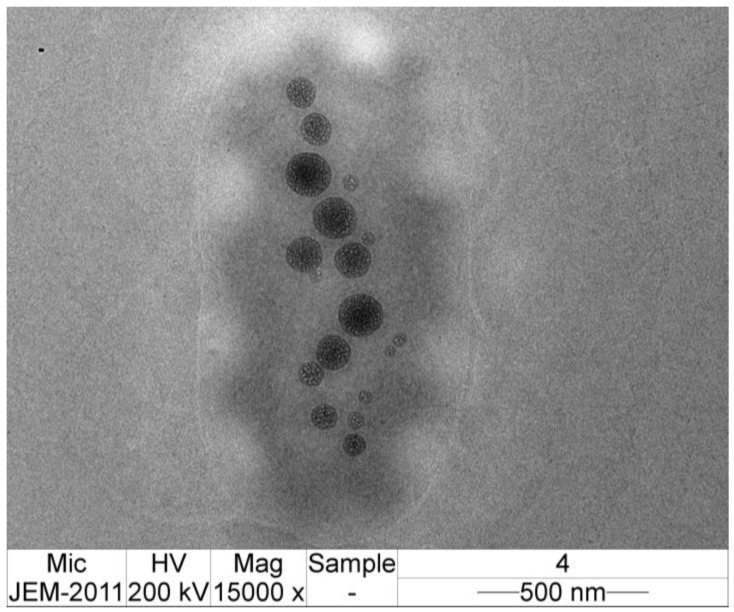
TEM imaging of CMCS-Ca^2+^ nanoparticles obtained from the ultrasound-assisted technique at 75% of amplitude and 180 s of time.

Figure 5 shows the TEM image of the nanoparticles obtained from the ultrasound-assisted technique at 75% of amplitude and 180 s of time. The TEM imaging showed that the nanoparticles were spherical in shape without conglomeration. The average particle diameter under TEM was 119.83 ± 41.19 nm, whereas the diameter from the Zetasizer was 318.40 ± 7.56 nm. The size of CMCS-Ca^2+^ nanoparticles from TEM was lower than that obtained using the Zetasizer, which may be because of the variation between different instruments.

### 3.7. SEM image of CMCS-Ca^2+^ Nanoparticles Film

**Figure 6:**
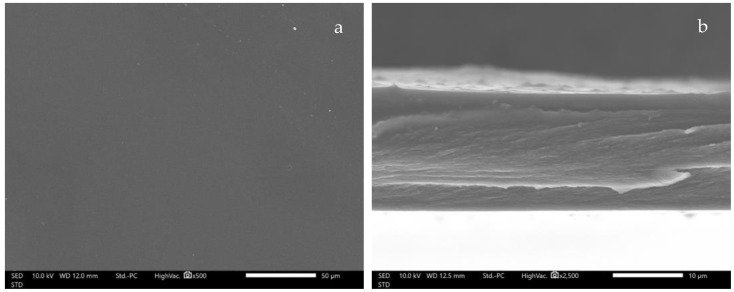

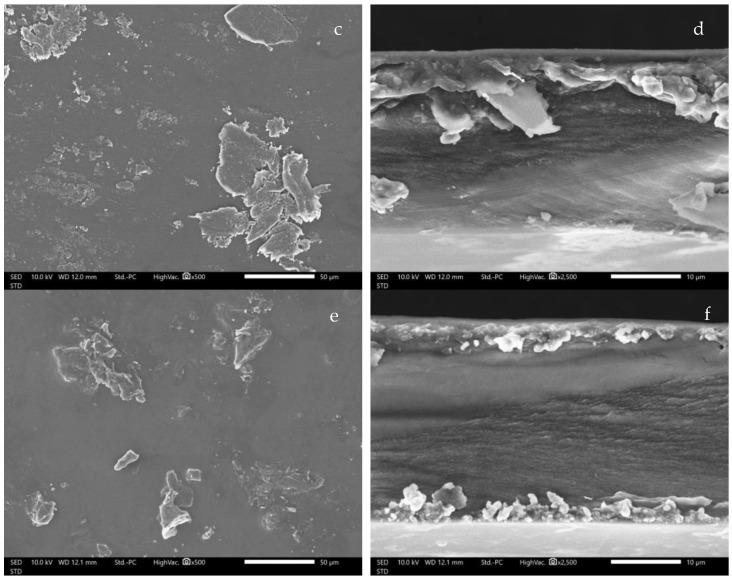
SEM micrographs of surface and cross-section of blank SCMC (**a**,**b**); SCMC-CM-powder (**c**,**d**); and SCMC-CM-nano films (**e**,**f**) at 500 and 2500 magnifications.

SEM micrographs of the SMCS films are shown in Figure 6. The blank SCMC film shows a smooth surface without any particles (Figure 6a). Likewise, the cross-sectional image shows a dense matrix. The SEM micrographs indicate the uniformity of the polymer matrix (Figure 6b). The thickness of the blank SCMC film was around 17 µm, whereas the thickness of the SCMC-CM-power and SCMC-CM-nano films was about 26 and 28 µm. The previous study also observed this characteristic in carboxymethyl cellulose (CMC) film [45]. The SEM micrographs of SCMC-CM-power and SCMC-CM-nano films exhibited an amorphous crystal structure of clindamycin HCl on the surface (Figure 6c,d). The cross-section images show rough texture (Figure 6e,f). These SEM images indicate that some part of clindamycin HCl in the polymer matrix were in the form of amorphous crystal structure. These results were also observed in pectin film containing clindamycin HCl [28]. However, the nanoparticles could not be observed in the SCMC-CM-nano films. Further investigations with TEM may reveal an internal structure of nanoparticle and/or polymer matrix of the fabricated films.

### 3.8. Clindamycin HCl Content in CMCS-Ca^2+^ Nanoparticles Film

The clindamycin HCl contents in SCMC-CM-powder and SCMC-CM-nano films were 100.81 ± 5.58% and 93.80 ± 9.24%, respectively. The high drug loading content could be explained by water solubility of the drug and polymer-drug interaction. Clindamycin HCl is a very water-soluble molecule dissolving in hydrophilic polymers such as SCMC in aqueous solution [28]. In the same way, CMCS is a hydrophilic polymer; thus the nanoparticles were compatible with SCMC and dispersed homogeneously in the polymer matrix. Moreover, the hydrophilic polymer could form hydrogen bonds with the hydrophilic groups on the drug molecule [19]. Hence, SCMC has abundant hydrophilic groups especially -OH and -COO^−^, which could form hydrogen bonds with -OH groups of clindamycin HCl.

### 3.9. Clindamycin HCl Release

**Figure 7:**
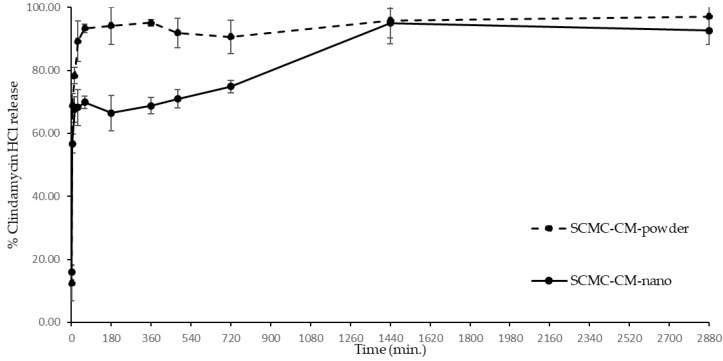
The release profile of clindamycin HCl power (SCMC-CM-powder) and clindamycin HCl in CMCS-Ca^2+^ nanoparticles (SCMC-CM-nano) from SCMC films.

The clindamycin HCl release profile, comparing clindamycin release patterns between clindamycin powder in SCMC film and clindamycin in CMCS-Ca^2+^ nanoparticles loaded in SCMC film, is shown in Figure 7. Basically, the extended release of antibacterial agents is important to maintain the level of the drug over MIC for a longer time. In topical applications, a film is one of the dosage forms that adhere to skin or mucous areas. In addition, the hydrophilic polymers, such as SCMC, immediately swell and release the drug, especially hydrophilic drugs, for example, clindamycin HCl, when the polymers have contact with water. Hence, the SCMC films were prepared to compare the drug release patterns of the clindamycin HCl in forms of free powder and nanoparticle encapsulation. SCMC-CM-powder exhibited a fast release of clindamycin HCl to 93.34 ± 1.29% within 60 min. The drug release percentage of SCMC-CM-powder escalated extremely from 12.39 ± 5.62% at 1 min to 93.34 ± 1.29% at 60 min. An accumulation of clindamycin HCl was clearly visible on the film surface (Figure 6c,d). Therefore, the release of clindamycin HCl might involve a superficial sequestering of clindamycin HCl located on the film surface and subsequent release of the drug from the SCMC matrix during the swelling of the polymer. After reaching the peak of release at 60 min, the percentage of the drug released was steady throughout the following 48 h. The fast drug release could be explained by the hydrophilic property of the drug. Clindamycin HCl is very soluble in water; thus it dissolves and releases immediately when the SCMC film comes into contact with water and swells [28]. Furthermore, SCMC and other compositions in the formulation, are hydrophilic in nature, which could swell and dissolve in water. In previous studies, more hydrophilic hydrogels exhibited higher swelling index and drug release [46,47]. It was possible that hydrophilic films, such as the SCMC films, released a high drug content immediately. The burst release of a hydrophilic drug from a hydrophilic polymeric film could be obviously delayed by polyelectrolyte complex technique. For example, chitosan/alginate polyelectrolyte complex film loading clindamycin phosphate showed the ability to delay the burst release of the hydrophilic drug. This is possibly due to a complexation between negative charged alginate (-COO^−^) and the positive charge of chitosan (-NH_3_^+^) and Ca^2+^ [48].

Meanwhile, the SCMC-CM-nano film exhibited different patterns of drug release when compared with the SCMC-CM-powder film. The first pattern of clindamycin release from the SCMC-CM-nano film increased from 15.91 ± 2.42% at 1 min to 69.88 ± 2.03% at 60 min. The burst release in this step related to the unencapsulated clindamycin HCl outside the nanoparticles. It is possible that higher nanoparticles containing more clindamycin HCl content might release slightly less drug content in this step. The burst release was also found in the previous study in which CMCS hydrogel containing clindamycin-loaded mesoporous silica nanoparticles showed initial burst release due to unentrapped clindamycin HCl that absorbed at the outer surfaces of particles and dispersed in the hydrogel matrix [49]. The initial drug release pattern of SCMC-CM-nano film was similar to the SCMC-CM-powder film. The second release step from 60 to 1440 min involved a gradual drug release from the nanoparticles in the SCMC-CM-nano film. It exhibited extended release and reached to 94.99 ± 4.70% at 1440 min. Generally, the drug release mechanism of cross-linked network structure is swelling-controlled release. The cross-linked network structure swells when it comes into contact with water, and then releases entrapped molecules inside the structure [50]. In the last release step, the level of clindamycin HCl was constant until 2880 min (48 h). The typical drug release from polymeric nanoparticle is driven by polymer degradation and/or drug diffusion [51]. It is possible that smaller particles, possessing a higher degradation rate, might demonstrate a faster release profile than larger particles. The pH of the media affected the drug release. The previous study stated that chitosan-based hydrogel exhibited higher drug release in an acidic medium than a neutral medium because the swelling of chitosan in an acidic medium is better than a neutral medium [52]. In this study, however, the experiment was performed in physiological pH (7.4), which is a general condition for the drug release profile and our study indicated that the SCMC-CM-nano films exhibited an extended drug release in this condition.

### 3.10. Antibacterial Activity

**Figure 8:**
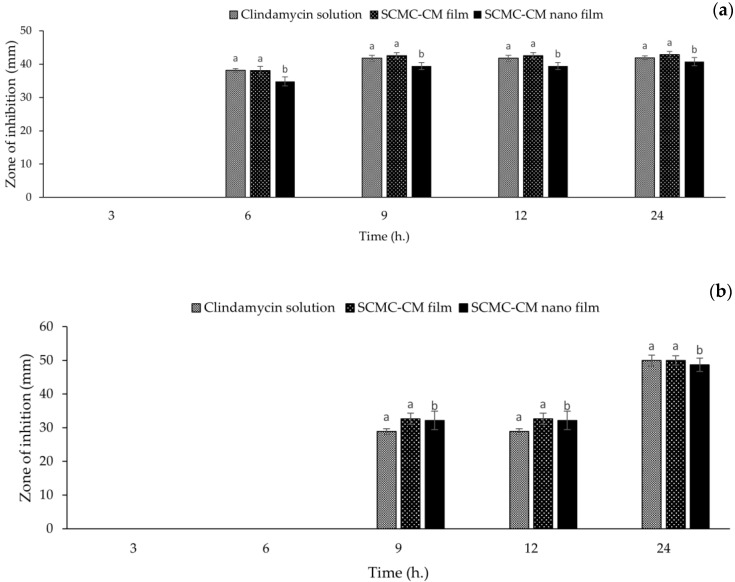
Antibacterial activity of clindamycin HCl solution, SCMC film containing clindamycin HCl powder (SCMC-CM-powder) and SCMC film containing clindamycin CMCS-Ca^2+^ nanoparticles (SCMC-CM-nano) against *S. aureus* (**a**) and *C. acnes* (**b**). Note: Different letters above the graphs indicate significant difference (*p* < 0.05).

The antibacterial activity of clindamycin HCl solution, SCMC-CM-powder film, and SCMC-CM-nano film against *S. aureus* and *C. acne* is shown in Figure 8. All of the samples contained the same amount of clindamycin HCl (1%). Overall, the zone of inhibition against *S. aureus* and *C. acne* increased with time. The blank SCMC film did not possess antibacterial activity against both of the tested bacteria because SCMC do not have any antibacterial activity [23]. Clindamycin HCl solution along with SCMC-CM-powder and SCMC-CM-nano films exhibited antibacterial activity because, generally, clindamycin HCl has the potential to inhibit the growth of *S. aureus* and *C. acne* [53]. In antibacterial activity against *S. aureus* (Figure 8a), the zone of inhibition of all of the samples were not observed at 3 h. The SCMC-CM-nano film showed the zone of inhibition as significantly lower (*p* < 0.05) than the clindamycin HCl solution and SCMC-CM-powder film at 6, 9, and 12 h. This might be the effect of the extended release of the SCMC-CM-nano film with a gradual release of the drug that was observed in the drug release profile (Figure 6). However, at 24 h, the zones of inhibition of the SCMC-CM-nano film increased to be significantly equivalent (*p* > 0.05) to the clindamycin HCl and SCMC-CM-powder film (40.72 ± 1.23, 41.81 ± 0.60 and 42.83 ± 0.97 mm, respectively). The zone of inhibition against *C. acne* was not generated in all of the samples at 3 and 6 h. The zone of inhibition of the SCMC-CM-nano film against *C. acne* was not significantly different from the clindamycin HCl solution and SCMC-CM-powder film throughout the experiment (Figure 8b). At 24 h, the clindamycin HCl solution, SCMC-CM-powder, and SCMC-CM-nano films exhibited insignificant differences (*p* > 0.05) of the zone of inhibition to each other (49.49 ± 1.65, 49.97 ± 1.41 and 48.70 ± 1.99 mm of the zone of inhibition, respectively). This result demonstrated antibacterial activity of the CMCS-Ca^2+^ nanoparticle containing clindamycin HCl in SCMC film. Thus, it could have potential use for an antibacterial application. In our study, however, SCMC-CM-nano films exhibit antibacterial activity similar to that of clindamycin solution. In contrast, the preparation of dual-cross-linked nanocomposite hydrogels based on quaternized chitosan and clindamycin-loaded hyperbranched nanoparticles showed that the dual-cross-linked nanocomposite hydrogels loading clindamycin exhibited better antibacterial activity than commercial clindamycin gel. This contrasting finding might be contributed to by positively-charged amino and quaternary ammonium groups of quaternized chitosan that target the bacterial cell wall [54].

## 4. Conclusions

CMCS nanoparticles loading clindamycin HCL as the model drug of water-soluble antibiotics were prepared by ionic interaction with Ca^2+^ with the ultrasound-assisted technique. The effect of intensity (amplitude) and duration (time) on the mean diameter of nanoparticles was investigated by experimental design. The model had the potential to predict the mean diameter of the produced nanoparticles. The study found that the size of the nanoparticles tended to decrease when amplitude and time increased. The obtained condition from the experimental design to obtain around 318 nm of nanoparticle size was 75% of amplitude and 180 s of time. The FTIR spectra showed that the peaks of carboxylic groups of CMCS in CMCS-Ca^2+^ nanoparticle shifted, and their intensity decreased. These indicated an interaction between the carboxylic groups of CMCS and Ca^2+^ to form polymeric nanoparticles. The TEM imaging showed that the CMCS-Ca^2+^ nanoparticles were in a spherical shape without aggregation. The clindamycin HCl loaded in a CMCS-Ca^2+^ nanoparticle was loaded into the SCMC film and exhibited 93.80 ± 9.24% of clindamycin HCl content. The drug release profile showed that the SCMC-CM-nano film performed an extended release pattern, compared with the release of clindamycin HCl powder loaded in SCMC film, which exhibited an immediate drug release pattern. Although the extended-release of SCMC-CM-nano film induced a slightly lower zone of inhibition against *S. aureus* at 6, 9, and 12 h, the SCMC-CM-nano film demonstrated good antibacterial activity against *S. aureus* and *C. acne* with 40.72 ± 1.23 and 48.70 ± 1.99 mm of the zone of inhibition at 24 h. These values were not significantly different from the clindamycin solution and SCMC-CM-powder film. Thus, this study indicated that CMCS-Ca^2+^ nanoparticles prepared by the ultrasound-assisted technique could be a potential carrier to modify the drug release of a small hydrophilic antibacterial molecule. For topical antibacterial application, however, further studies, such as in vitro cell cytotoxicity and irritation tests, should be investigated.

## Figures and Tables

**Table 1:** Coded experimental level of each variable factor.

Variables	Level				
	−α	−1	0	+1	α
Amplitude (%)	31.71	40	60	80	88.28
Time (s)	10.29	60	180	300	349.71

**Table 2:** ANOVA of quadratic model.

Source	Sum of Squares	Mean Square	F-Value	*p*-Value
Model	39,098.83	7819.77	131.77	<0.0001
A-Amplitude	23,502.49	23,502.49	396.04	<0.0001
B-Time	4571.19	4571.19	77.03	<0.0001
AB	73.10	73.10	1.23	0.3037
A^2^	8109.88	8109.88	136.66	<0.0001
B^2^	4173.52	4173.52	70.33	<0.0001
Residual	415.41	59.34		
Lack of Fit	148.45	49.48	0.7414	0.5804
Pure error	266.96	66.74		

**Table 3:** Variable levels and responses of particle sizes based on amplitude and time.

Exp. Order	Variables		Response		PDI
			Mean Diameter (nm)		
	Amplitude (%)	Time (s)	Actual Value	Predicted Value	
1	40	60	478.40 ± 4.12 *	477.57 *	0.442 ± 0.025
2	80	60	384.20 ± 14.38 *	377.71 *	0.515 ± 0.094
3	40	300	432.70 ± 4.89 *	438.31 *	0.456 ± 0.093
4	80	300	321.40 ± 15.03 *	321.36 *	0.394 ± 0.040
5	31.72	180	493.57 ± 5.76 *	490.04 *	0.594 ± 0.147
6	88.28	180	323.30 ± 8.40 *	336.73 *	0.372 ± 0.044
7	60	10.29	422.87 ± 17.42 *	427.89 *	0.439 ± 0.101
8	60	349.71	364.42 ± 16.80 *	360.28 *	0.422 ± 0.065
9	60	180	341.11 ± 13.72 *	345.10 *	0.551 ± 0.106
10	60	180	333.50 ± 6.45 *	345.10 *	0.513 ± 0.185
11	60	180	347.31 ± 15.97 *	345.10 *	0.455 ± 0.042
12	60	180	355.12 ± 5.54 *	345.10 *	0.407 ± 0.011
13	60	180	348.47 ± 10.08 *	345.10 *	0.328 ± 0.030
Test data					
14	50	150	394.30 ± 4.50 *	387.71 *	0.316 ± 0.057
15	65	200	335.90 ± 6.71 *	330.20 *	0.236 ± 0.025
16	75	180	318.40 ± 7.56 *	323.65 *	0.289 ± 0.064
17	50	80	421.22 ± 10.45 *	415.89 *	0.489 ±0.164

Note: S.D. of predicted value was 7.70. * The same superscript symbol in the same row indicates insignificant different (*p* > 0.05) by *t*-test.

**Table 4:** Clindamycin loading content in CMCS-Ca^2+^ nanoparticles in each experiment.

Exp. Order	Mean Diameter (nm)	Drug Content (%)
1	478.40 ± 4.12 ^a^	46.88 ± 4.86 *
2	384.20 ± 14.38 ^b^	41.45 ± 2.60 ^#◊^
3	432.70 ± 4.89 ^c^	47.66 ± 3.51 *
4	321.40 ± 15.03 ^d^	33.30 ± 2.04 ^†^
5	493.57 ± 5.76 ^a^	46.52 ± 2.91 *
6	323.30 ± 8.40 ^df^	34.44 ± 1.71 ^†^
7	422.87 ± 17.42 ^c^	41.63 ± 1.53 ^#∆◊^
8	364.42 ± 16.80 ^bhk^	31.93 ± 3.20 ^†^
9	341.11 ± 13.72 ^di^	34.26 ± 3.34 ^†^
10	333.50 ± 6.45 ^djm^	33.12 ± 1.77 ^†^
11	347.31 ± 15.97 ^fhijk^	33.54 ± 1.63 ^†^
12	355.12 ± 5.54 ^djkl^	31.63 ± 2.55 ^†^
13	348.47 ± 10.08 ^hilmn^	40.03 ± 2.91 ^∆◊^
14	394.30 ± 4.50 ^b^	31.99 ± 2.75 ^†^
15	335.90 ± 6.71 ^djn^	34.50 ± 1.17 ^†^
16	318.40 ± 7.56 ^dj^	34.68 ± 2.45 ^†^
17	421.22 ± 10.45 ^c^	41.02 ± 2.27 ^#◊^

Note: Different superscript letters and symbols in the same columns of mean diameter and drug content, respectively indicate significant difference (*p* < 0.05) between each Exp. order.

## Data Availability

Not applicable.
